# Zinc Oxide Nanoparticles Enhanced Biomass and Zinc Content and Induced Changes in Biological Properties of Red *Perilla frutescens*

**DOI:** 10.3390/ma14206182

**Published:** 2021-10-18

**Authors:** Piotr Salachna, Małgorzata Mizielińska, Beata Płoszaj-Witkowska, Agnieszka Jaszczak

**Affiliations:** 1 Department of Horticulture, West Pomeranian University of Technology in Szczecin, Papieża Pawła VI 3, 71-459 Szczecin, Poland; 2 Center of Bioimmobilisation and Innovative Packaging Materials, West Pomeranian University of Technology in Szczecin, Janickiego 35, 71-270 Szczecin, Poland; malgorzata.mizielinska@zut.edu.pl; 3 Department of Landscape Architecture, University of Warmia and Mazury, 10-719 Olsztyn, Poland; beata.ploszaj@uwm.edu.pl (B.P.-W.); agnieszka.jaszczak@uwm.edu.pl (A.J.)

**Keywords:** nanofertilizers, nanotechnology, medicinal plants, antioxidants, antibacterial activity

## Abstract

The above-ground parts of plants, including leaves, constitute an important part of a human diet. Their mineral and biological composition can be modified by proper preparation of the soil substrate, i.e., supplying it with nutrients. The objective of this study was to assess the influence of zinc oxide nanoparticles (ZnO NPs) at 0, 50, 100 and 200 mg L^−1^ concentrations on red perilla (*Perilla frutescens* var. *crispa* f. *purpurea*) leaf yield and quality. Plants were grown in 2 L pot under a plastic greenhouse condition. The exposure to ZnO NPs increased leaf fresh and dry weight and leaf Zn content as compared with untreated control. Fresh weight boost was the most pronounced at 50 mg L^−1^ ZnO NPs. The lowest concentration of ZnO NPs also enhanced the content of total polyphenols, antioxidant activity, and antiradical activity. Treatments with 50 or 100 mg L^−1^ ZnO NPs boosted the level of total anthocyanins and bacteriostatic activity of 25% extracts. Overall, this study demonstrated that ZnO NPs at low rates is useful as a biostimulant and nanofertilizer for red perilla production.

## 1. Introduction

Nanotechnology is a branch of science offering the possibility of obtaining structures, forms of elements and compounds of nano-size (1 to 100 nm) that usually show very high chemical and biological activity. Among the engineered nanomaterials applied in agriculture and horticulture, the most interesting seem to be the nanoparticles of metal oxides used in the new generation of agrochemicals as fertilizers, plant protection products, herbicides and biostimulants [1,2,3]. Metal nanoparticles can positively influence plant growth and flowering [4,5], enhance crop productivity [6,7], and improve plant resistance to stress [8,9]. On the other hand, numerous studies have demonstrated possible phytotoxic effects of nanoparticles on plants, including disturbed stability of physiological and biochemical processes [10]. Wide interest of researchers in agricultural practices and still inconclusive results spur on intensive research aimed at better understanding of plant response to nanoparticles of metal oxides.

The problem of zinc deficiency still persists and expands to new areas [11]. Zinc plays a crucial role in the daily functioning of the human body. It is necessary for the proper growth and regeneration of tissues, affects the structure of some proteins, the absorption of vitamins (vitamin A), and is responsible for the appropriate functioning of the immune system [12]. The sources of zinc in the diet include, e.g., products of plant origin, but plant-derived zinc is less effectively assimilated. Zinc content in edible plant parts can be considerably heightened by using fertilizers containing available zinc forms in the amounts exceeding those required for optimal plant growth [13,14].

Zinc is an indispensable plant micronutrient controlling the activity of numerous enzymes and hormones, regulating the metabolism of macromolecules, stabilizing protein structures and controlling gene expression [15]. Both deficiency and excess of zinc are harmful to plants, and its effects depend on its form, solubility and factors affecting the solubility [11]. Zinc bioaccessibility in soils is often poor, as it binds to insoluble compounds [10]. This problem can be solved by using fertilizers in the form of salts, chelates and complexes. However, zinc applied this way is poorly utilized by plants. A viable alternative may be nanofertilizers containing zinc in the form of zinc oxide nanoparticles (ZnO NPs) that may be more effectively absorbed by plants as showed for coffee [16], rice [4] and wheat [17]. Other positive effects of plant treatment with ZnO NPs include stimulation of germination and seedling emergence, increase of biomass accumulation, net photosynthetic rate, pigment content, or nutrient uptake. The final effects depend on the species, concentration and application method [18,19,20]. A few papers have indicated that ZnO NPs can also stimulate or inhibit metabolite biosynthesis and modify antioxidant activity [21,22,23]. The last trait seems particularly interesting in the case of herbal and medicinal plants.

Perilla (*Perilla frutescens* (L.) Britton), a member of the Lamiaceae family, has for centuries been used as a fragrant and medicinal herb. The species spread through Japan to other Asian countries and nowadays it is widely cultivated in the United States, North and South Africa and Europe, with notable economic returns [24,25,26]. In Japan, Korea, Thailand, Vietnam and India it is a very popular culinary herb and aromatic vegetable due to its aroma, basil-like taste and nice colors, while in China—it is mainly used as a medicinal plant [27]. The extracts of perilla were reported to have antiviral, antimicrobial, antioxidant, anti-inflammatory, anti-allergic, anti-asthmatic, anti-diabetic, anti-depressant, anti-aging and anti-cancer activities [24,25,26,27]. Red perilla has dark purple foliage used in food as a natural colorants and good source of antioxidants [28]. Apart from its functional properties, red perilla is a highly attractive ornamental plant grown in many areas of the world in gardens and flower beds. New technologies of cultivation and fertilization are constantly being sought for to provide perilla with optimal growing conditions and obtain high quality yield [29]. Zhang et al. [30] demonstrated that treating the seeds of *P. frutescens* with zinc sulfate (ZnSO_4_) effectively improved the germination rate and antioxidant activity of the seedlings. The effects of zinc fertilization on productivity and biological value of perilla have not been investigated so far.

Therefore, the aim of this study was to assess the yield, leaf content of zinc and secondary metabolites, as well as antioxidant and bacteriostatic activity of red perilla leaf extracts following treatment with variable doses of zinc applied as ZnO NPs. To avoid possible phytotoxic effects of ZnO NPs on the aerial plant parts, ZnO NPs was applied by drenching. We assumed that ZnO NPs may improve yield and content of bioactive compounds in perilla leaves.

## 2. Materials and Methods

### 2.1. Preparation of ZnO NPs

The study used ZnO NPs of the size ~70 nm. The particle size and distribution of ZnO NPs were measured using Mastersizer 2000 (Malvern, UK). ZnO NPs (0.1 g) were introduced into 99.9 mL of distilled water. As the first step, the solution was mixed for 1 h using a magnetic stirrer (450 rpm, Ika, Staufen, Germany). Next, it was sonicated (sonication parameters: cycle: 0.5; amplitude: 20%; time: 10 min). Then, the solution was stirred for 15 min with a magnetic stirrer (450 rpm). The stock solution was serially diluted with water to obtain ZnO NPs solution with the concentration of 50, 100 and 200 mg L^−1^.

### 2.2. Plant Material and Experimental Conditions

The seeds of red perilla (*P. frutescens* var. *crispa* f. *purpurea*) were sown on 15 March 2018 into seed trays kept in a greenhouse with day/night air temperatures of 22/18 °C. After six weeks the seedlings matched for size were individually transferred into 2 L black pots filled with substrate based on sphagnum peat moss (pH 6.5) mixed with a fertilizer (Yara International ASA, Oslo, Norway) (12% N, 4.8% P, 14.9% K, 1.6% Mg, 8% S, 0.015% B, 0.2% Fe, 0.02% Mn, and 0.02% Zn) at 3 g L^−1^. The plants in the pots were placed in a completely randomized design in a plastic greenhouse and grown under natural photoperiod. Each experimental group comprised 40 plants, 10 plants per repetition. The mean minimum and maximum air temperatures through the growing season fluctuated between 12.0–15.8 °C and 29.2–31.8 °C, respectively. When the plants reached 30–40 cm, zinc fertilization was applied as aqueous solutions of ZnO NPs. Each pot was drenched three times, every five days, and a total of 300 mL of the solution per pot were used. Control plants were provided with water.

### 2.3. Determination of Fresh and Dry Weight

On 28 August 2018 (169 days after sowing) all leaves were collected individually from five matched plants from each repetition (total 20 plants) and their fresh weight was determined using an electronic scale. From each of the five matched plants from each repetition 10 fully expanded leaves were collected from the upper and middle part of the plant. The leaves were rinsed with water and dried on paper for four weeks, in darkness, at 25–30 °C. Leaf dry weight was determined by drying to a constant weight at 105 °C [31].

### 2.4. Determination of Zn Concentration

Zinc content in leaf tissues was determined by atomic absorption spectrophotometry [32], in samples mineralized for 8 h in a mixture of HNO_3_ and HClO_4_ (1:4), using 30 mL of the mixture per 2 g of dry plant material. The Zn content was expressed in mg kg^−1^ dry weight (DW).

### 2.5. Determination of Total Anthocyanin Concentration

The extraction procedure followed the method [33]. The anthocyanin assay was carried out according to our previous paper [34]. The total anthocyanin content was expressed as grams of cyanidin-3-glucoside (C3G) 100 g^−1^ DW.

### 2.6. Determination of Total Polyphenol Content and Antioxidant Capacity Assays

The preparation of plant extracts followed the method [35] with some modifications according to our previous report [34]. Total polyphenol content was analyzed spectrophotometrically using the Folin–Ciocalteu colorimetric method [35]. Gallic acid was used to calculate the standard curve, and the results were expressed as GAE milligrams per g of DW. Determination of DPPH (2,2-diphenyl-1-picrylhydrazyl) radical scavenging capacity of perilla leaves followed the method [36]. The ferric reducing antioxidant power (FRAP) assay was carried out according to our previous report [37]. The results of DPPH and FRAP assays were expressed as mg trolox equivalents (TE) g^−1^ DW.

### 2.7. Antibacterial Activity Assays

*Staphylococcus aureus* DSMZ 346 and *Escherichia coli* DSMZ 498 were used as test microorganisms. They were purchased from the collection of the Leibniz Institute DSMZ (Braunschweig, Germany). To verify antibacterial activity of the prepared extracts, TSB, TSA and MacConkey agar (Merck, Darmstadt, Germany) were used. All media were prepared according to the Merck protocol. As next step, ten grams of dried leaf tissue from the plants drenched with 0, 50, 100 or 200 mg L^−1^ ZnO NPs were extracted with 100 g of 70% aqueous acetone. The aqueous acetone solutions containing the dried leaf samples were kept in a sonication bath for 1 h, at a constant 15 °C by adding ice to the water. The sonification parameters were: cycle—0.5; amplitude—50%. The crude acetone extracts were filtered through a Büchner funnel containing cellulose filter. Then the 70% aqueous acetone extracts were concentrated at 40 °C. After acetone evaporation, the samples were filtered through a 0.2 µm filter and used in the next experiments. The antibacterial analysis was performed using the methods described in our previous paper [34]. As first step of the antibacterial analysis, the *S. aureus* cells were pre-grown on TSA and *E. coli* cells were pre-grown on MacConkey agar for 24 h at 30 °C. After incubation, the biomass (each strain separately) was suspended in a sterile 0.85% NaCl solution to obtain 1.5 × 10^8^ CFU/mL. The next step was to prepare 25% and 50% solutions of perilla extracts in 10 mL of TSB medium in flasks. The samples were mixed with a magnetic stirrer (250 rpm, Ika) for 15 min. Then the mixture of the extracts and TSB were introduced into the test tubes. The suspended biomass of microorganisms (100 µL of each sample) was added to sterile test tubes, which contained TSB with extracts at a concentration of 25%, 50%, and further was mixed with a vortex (Ika, Legnica, Poland) for 1 min. The medium containing biomass that did not contain any extracts was the control sample. After stirring, 100 µL of each sample was plating on TSA and MacConkey agar and incubated at 30 °C for 24 h. The cell concentration was expressed as colony-forming units (CFU) per mL. All tests were performed in triplicates.

### 2.8. Statistical Analysis

All laboratory analyses were performed in three replications, and the final means are presented with their standard errors (SEM). To verify if ZnO NPs dose as an independent variable affected the study results, one way ANOVA was performed following prior testing of the normality of the distribution of dependent variables with TIBCO Statistica^®^ 13.3 (StatSoft Poland) software. The comparison of the final means and determination of homogeneous groups were performed using the Tukey’s test.

## 3. Results and Discussion

The issues created by zinc deficiency in food are due, inter alia, to low bioavailability of this micronutrient in the soil and, consequently, its insufficient content in plant products [7,13,14]. Our study is the first to investigate the effects of fertilization with zinc nanoparticles on leaf yield of an edible and medicinal plant red perilla.

**Figure 1:**
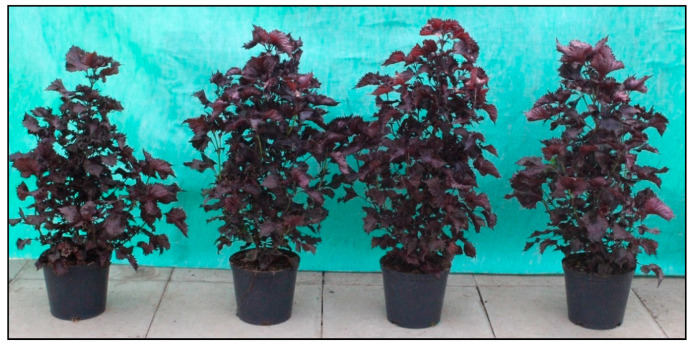
Substrate drench effects of zinc oxide nanoparticles (ZnO NPs) on growth red perilla. Treatments are left to right: water (control), 50, 100, and 200 ZnO NPs mg L^−1^.

Table 1 shows clear stimulating effects of soil application of ZnO NPs on fresh and dry weight of red perilla leaves. Irrespective of ZnO NPs concentration, the plants fertilized with zinc reached greater fresh and dry weight of leaves, by on average 29.5% and 18.9%, versus controls not treated with ZnO NPs. The increase in fresh and dry weight as a result of ZnO NPs application was also reported in carrot [20], rice [19] and tomato [22]. The beneficial effects of ZnO NPs on the biomass may be related to the positive effect of zinc on the mineral balance and better nutrient supply status of the plants. The extent of the biostimulation was dose dependent. The greatest fresh and dry weight of leaves was determined for the plants drenched with 50 mg L^−1^ ZnO NPs solution. Lower doses of ZnO NPs (up to 50 mg kg^−1^) applied to the soil are more beneficial than higher doses, as confirmed by research studies [7,23]. High levels of ZnO NPs can be phytotoxic and limit plant yield [38]. Therefore, to achieve optimal biomass growth it is important to supply plants with low doses of zinc NPs, similarly as in the case of other micronutrients. As shown in Figure 1, the plants showed no phytotoxic effects of ZnO NPs with 50–200 mg L^−1^. It is also worth emphasizing that the higher leaf biomass of plants treated ZnO NPs can improve red perilla aesthetic value. Many cultivars of perilla are very attractive ornamental plants widely used in the landscape.

One of the strategies for optimizing the diet is to enrich food with deficient nutrients in order to improve the nutritional status and health of various population groups [13]. A popular method for counteracting micronutrient deficiency in humans is feeding plants with fertilizers containing ingredients in a more available form; e.g., of nanoparticles. In this context, our results confirming a significant (24.6–40.5%) increase of zinc content in the leaves of red perilla treated with ZnO NPs seem highly interesting (Table 2). The leaves of the plants drenched with ZnO NPs at 200 mg L^−1^ reached the highest zinc content. Similar outcomes were achieved in earlier studies in beans and tomatoes grown in the soil enriched with 3, 20 or 225 mg ZnO NPs kg ^−1^ [38]. Again, the leaves of the plants treated with the highest dose of ZnO NPs accumulated the greatest amounts of the metal.

A large proportion of plant polyphenols show strong antioxidant activity, which is responsible for many beneficial biological effects of plant products. Polyphenol-rich species attract particular attention of consumers for their increased pro-health value. Our findings on the total polyphenol content in red perilla (control plants—59.37 mg GAE g^−1^ DW, Table 2) were close to 57.67 mg GAE g^−1^ DW reported by Chou et al. [39] and lower than 93.7 mg GAE g^−1^ DW recorded by Rouphael et al. [26]. On the other hand, in a study on the stalks and leaves of red perilla [40] detected lower amounts of total polyphenols (22.67 mg GAE g^−1^ DW), which probably was, as mentioned in the paper, due to the extraction method. It was also found that phenolic acid content varied significantly with the genotype of perilla, its growing stage, environmental factors [27], and biotic and abiotic elicitors [26].

The total content of polyphenols in the leaves of red perilla was similar in the treated and untreated variants, but some differences were found depending on the concentration of ZnO NPs (Table 2). Total polyphenols were more abundant in the leaves of plants treated with 50 mg L^−1^ than 200 mg L^−1^ ZnO NPs. Our results are partially in line with the results of other studies showing multi-directional changes in the content of non-enzymatic antioxidants depending on the dose of ZnO NPs [23,41].

Among phenolic compounds found in plants, flavonoids are the most widely studied class of polyphenols with respect to their antioxidant activities. One of the largest flavonoid groups responsible for red, purple and blue color of fruits, vegetables and flowers are anthocyanins (cyanidin, delphinidin, pelargonidin, peonidin, malvidin and petunidin) [42]. Studies on anthocyanin contents in *P. frutescens* var. *crispa* revealed that red color depended on the presence of a cyanidin-type anthocyanin, 3-*O*-(6-*O*-(E)-p-coumaryl-β-d-glucopyranosyl)-5-*O*-(6-*O*-malonyl-β-d-glucopyranosyl)-cyanidin, malonylshisonin [43]. Our study assessed the leaves of red perilla for their content of total anthocyanins—a group of polyphenols well known for their antioxidant properties. Our analysis confirmed its levels in the control plants to reach 1.62 g C3G 100 g^−1^ DW (Table 2). Hwang [44] reported that anthocyanin content in the leaves of *P. frutescens* var. *crispa* was 2.3 g 100 g^−1^ DW, while in the leaves of *P. frutescens* var. *acuta* it was 1.2–1.7 g 100 g^−1^ DW. Plant treatment with ZnO NPs at 50 and 100 mg L^−1^ clearly enhanced total anthocyanin content as compared with the control (by 35.8% and 34.0%, respectively). Numerous studies showed that metal nanoparticles may act as elicitors that stimulate the production of secondary plant metabolites [45].

As previously mentioned, the raw materials of perilla are a valuable source of biologically active compounds, including ingredients with antioxidant potential. We assessed the antioxidant capacity of the extracts using methods based on chemical reactions between an antioxidant and a model free radical (DPPH) or Fe^3+^ (FRAP) (Table 3). In the control plants, DPPH values reached 138.10 mg TE g^−1^ DM, and for FRAP 29.98 mg TE g^−1^ DM. Assefa et al. [46] examined dried extracts of the leaves in 73 accessions of green pigmented landrace varieties of *P. frutescens*, and DPPH radical scavenging activities varied from 16.54 to 74.35 mg AAE g^−1^. Kim et al. [47], who measured the antioxidant potential of leaf extracts of *P. frutescens* var. *japonica* reported EC_50_ = 407 μg DW mL^−1^ for DPPH and 2.32 mmol FeSO_4_ · 7 H_2_O g^−1^ DW for FRAP. In our study, ZnO NPs at 50 and 100 mg L^−1^ significantly enhanced plant capacity to neutralize free DPPH radical as compared with the untreated control. The ferric-reducing antioxidant power of leaf extracts of red perilla was the greatest in the plants treated with 50 mg L^−1^ ZnO NPs. Improved antiradical activity was probably due to elevated content of anthocyanins.

Perilla extracts may show antibacterial activity, which is particularly important in the search for bioactive plant substances that can be used to fight pathogens, especially multi-drug resistant ones. Our study confirmed bacteriolytic effects of all 50% leaf extracts of red perilla toward both *S. aureus* and *E. coli*, as no bacilli or cocci were detected after 24 h incubations with the extracts (Table 4). Kang et al. [48] also demonstrated antibacterial activity of perilla extracts toward gram-positive and gram-negative bacteria. Similarly, Yamamoto and Ogawa [49] reported that ethanol and ethyl acetate extracts of perilla inhibited growth of *Streptococcus* spp. Another study [50] showed growth inhibition of *E. coli* strains treated with perilla extracts. The antibacterial activity of the extracts was probably due to phytochemicals, including essential oils of perilla leaves that inhibit growth of many bacteria [24].

In our study, the tested concentrations of ZnO NPs did not affect the antibacterial activity of 50% extracts, but we noticed some differences for 25% extracts (Table 4). A comparison of *S. aureus* count revealed the greatest (by four logarithmic orders) decrease after incubation with the extracts obtained from the plants treated with 100 mg L^−1^ ZnO NPs. Moreover, following treatment of the plants with 50 mg L^−1^ ZnO NPs, we also observed a 4-log reduction in *S. aureus* count after the incubation with 50% extracts. The highest concentration of ZnO NPs (200 mg L^−1^) negatively affected the antimicrobial properties of the extracts against gram-positive cells, as the reduction of *S. aureus* count was about an order lower than for the extracts obtained from plants treated with lower concentrations of ZnO NPs. The extracts obtained from the plants treated with 50 and 200 mg L^−1^ ZnO NPs showed weaker anti *E. coli* activity than those from the untreated controls. The extracts most effective against the gram-negative bacilli were those derived from plants exposed to 100 mg L^−1^ ZnO NPs. Summing up the microbiological analyses, we concluded that 50% leaf extracts of red perilla completely inhibited the growth of *S*. *aureus* and *E. coli*, while 25% extracts slowed it down considerably.

## 4. Conclusions

Our experiments demonstrated improvement in the quality and quantity of red perilla leaf yield as a result of drenching the plants with ZnO NPs. The effects were dose dependent. Lower concentrations of ZnO NPs (50 and 100 mg L^−1^) increased plant biomass, total anthocyanin content, antiradical and bacteriostatic activity of 25% extracts, while the highest concentration (200 mg L^−1^) reduced their antimicrobial activity. We also found growing Zn content in the leaves along with rising concentrations of ZnO NPs. The benefits of applying low doses of ZnO NPs in the cultivation of red perilla are undeniable, but due to the controversies regarding the impact of nano-scale materials on living organisms, further research is needed to assess the safety of ZnO NPs release into the environment.

## Figures and Tables

**Table 1:** Effects of zinc oxide nanoparticles (ZnO NPs) on fresh and dry weight content of red perilla leaves.

Treatment	Fresh Weight of Leaves per Plant (g)	Dry Weight of Leaves (%)
Control	93.9 ± 1.94 c *	19.0 ± 0.21 c
50 mg L^−1^ ZnO NPs	121.6 ± 2.58 a	22.6 ± 0.35 a
100 mg L^−1^ ZnO NPs	101.8 ± 2.69 b	20.4 ± 0.26 b
200 mg L^−1^ ZnO NPs	103.7 ± 2.54 b	21.1 ± 0.20 b

* Values are mean ± SEM for *n* = 3. Values within a column marked with the same letter are not significantly different at *p* ≤ 0.05 based on Tukey’s test.

**Table 2:** Effects of zinc oxide nanoparticles (ZnO NPs) on concentration of zinc, total polyphenols and total anthocyanins in red perilla leaves.

Treatment	Zinc Content (mg kg^−1^ DW)	Total Polyphenols (mg GAE g^−1^ DW)	Total Anthocyanins (g C3G 100 g^−1^ DW)
Control	45.17 ± 2.78 c *	59.37 ± 0.18 ab	1.62 ± 0.13 b
50 mg L^−1^ ZnO NPs	56.27 ± 3.88 b	63.30 ± 2.05 a	2.20 ± 0.13 a
100 mg L^−1^ ZnO NPs	58.07 ± 1.99 b	61.74 ± 2.12 ab	2.17 ± 0.21 a
200 mg L^−1^ ZnO NPs	63.47 ± 1.21 a	56.41 ± 2.12 b	1.62 ± 0.15 b

* Values are mean ± SEM for *n* = 3. Values within a column marked with the same letter are not significantly different at *p* ≤ 0.05 based on Tukey’s test.

**Table 3:** Effects of zinc oxide nanoparticles (ZnO NPs) on antiradical activity (DPPH) and antioxidant activity (FRAP) of red perilla leaves.

Treatment	DPPH (mg TE g^−1^ DW)	FRAP (mg TE g^−1^ DW)
Control	138.10 ± 1.88 b *	29.98 ± 0.08 ab
50 mg L^−1^ ZnO NPs	145.83 ± 1.37 a	30.14 ± 0.11 a
100 mg L^−1^ ZnO NPs	147.40 ± 2.01 a	30.03 ± 0.02 ab
200 mg L^−1^ ZnO NPs	130.67 ± 2.11 c	29.86 ± 0.08 b

* Values are mean ± SEM for *n* = 3. Values within a column marked with the same letter are not significantly different at *p* ≤ 0.05 based on Tukey’s test.

**Table 4:** Antibacterial activity of red perilla extracts from plants treated with zinc oxide nanoparticles (ZnO NPs).

Treatment	Extract Concentration (%)	The Number of Bacterial Cells (10^5^ CFU/mL)	
		*S. aureus*	*E. coli*
Control	50	0	0
	25	5.50 ± 0.71 b *	2.50 ± 0.57 b
50 mg L^−1^ ZnO NPs	50	0	0
	25	2.60 ± 0.99 c	4.20 ± 0.01 a
100 mg L^−1^ ZnO NPs	50	0	0
	25	2.05 ± 0.78 c	1.20 ± 0.71 c
200 mg L^−1^ ZnO NPs	50	0	0
	25	16.60 ± 2.33 a	4.25 ± 0.15 a
The number of bacterial cells for positive control (10^9^ CFU/mL)		1.28 ± 0.04	6.00 ± 1.70

* Values are mean ± SEM for *n* = 3. Logarithm values within a column marked with the same letter are not significantly different at *p* ≤ 0.05 based on Tukey’s test.

## Data Availability

Data is contained within the article.
